# Effectiveness of AI-Assisted Digital Therapies for Post-Stroke Aphasia Rehabilitation: A Systematic Review

**DOI:** 10.3390/brainsci15091007

**Published:** 2025-09-18

**Authors:** Yamil Liscano, Lina Marcela Bernal, Jhony Alejandro Díaz Vallejo

**Affiliations:** 1 Grupo de Investigación en Salud Integral (GISI), Departamento Facultad de Salud, Universidad Santiago de Cali, Cali 760035, Colombia; 2 Grupo de Investigación en Fonoaudiología y Psicología, Facultad de Salud, Universidad Santiago de Cali, Cali 760035, Colombia; lina.bernal02@usc.edu.co; 3 Basic Health Sciences Department, Research Group on Nutrition, Metabolism and Food Safety, University of Caldas, Manizales 170004, Colombia; alejandrodiazval@gmail.com

**Keywords:** aphasia, stroke, artificial intelligence, digital health, rehabilitation, systematic review

## Abstract

**Background:** Traditional aphasia therapy is often limited by insufficient dosage, a barrier that AI-assisted digital therapies are poised to overcome. However, it remains unclear whether gains on specific tasks translate to functional, real-world communication. This systematic review evaluates the effectiveness of these novel interventions and investigates the potential for a “generalization gap” when compared to conventional treatments for post-stroke aphasia rehabilitation. **Methods:** Following PRISMA guidelines, we systematically reviewed randomized controlled trials (2010–2024) from six databases. We included studies examining AI-powered digital platforms for adults with chronic post-stroke apha-sia that reported standardized language outcomes. **Results:** Our analysis of five trials (*n* = 366) shows that AI-assisted therapies successfully deliver high-dose interventions, leading to significant improvements in trained language skills, including word retrieval (up to 16.4% gain) and auditory comprehension. However, a critical “generalization gap” was consistently identified: these impairment-level gains rarely transferred to functional, real-world communication. **Conclusions:** AI-assisted digital therapies effectively solve the dosage problem in aphasia care and improve specific linguistic deficits. Their primary limitation is the failure to generalize skills to everyday use. Future platforms must therefore be strategically redesigned to incorporate therapeutic principles that explicitly target the transfer of skills, bridging the gap between clinical improvement and functional communication.

## 1. Introduction

Aphasia is an acquired language disorder that affects the brain areas responsible for expression and comprehension in communication [1]. Among its most frequent etiologies is stroke (cerebrovascular accident or CVA), and it is estimated that approximately 31.4% of people who survive a stroke will experience language sequelae [2]. This directly affects the person’s quality of life and social interaction, leading to a restructuring of their daily functions. The Global Burden of Diseases, Injuries, and Risk Factors Study (GBD) shows that the global incidence of stroke in 2021 was 11.9 million new cases and is the fourth leading cause of disability worldwide [3].

The type of post-stroke aphasia will depend on the neuroanatomical location of the lesion and is usually associated with damage to the left hemisphere. In addition, the profile of damage depends on multiple factors such as the time elapsed after stroke and the specific location of the lesion [1]. Broadly, two major categories are recognized that have important classificatory features. Non-fluent aphasias are characterized by sparse speech, agrammatism, and relatively preserved comprehension, associated with left frontal lesions. In contrast, fluent aphasias, secondary to left temporoparietal lesions, manifest with abundant but paragrammatic language, severe comprehension deficits, and phonemic paraphasias [4].

Language rehabilitation is a clinical priority, as restoring language functions requires neural changes to reorganize information through cerebral neuroplasticity [5] and these mechanisms facilitate learning processes and behavioral readaptation. The goal of speech-language therapy is to optimize linguistic abilities and social participation via functional communication, with a particular focus on language production and comprehension [6]. However, the optimal timing to initiate intervention and its duration remain controversial, as they depend on multiple factors, including the patient’s immediate context, individual variability in cognitive reserve, and the specificity of interventions [7,8].

Several traditional approaches focus on picture naming, action description, and auditory discrimination through linguistic stimulation and structured repetition in in-person sessions between the therapist and patient [9]. The sequentiality of treatment is based on individual adaptation to the specific needs of each person and continuous monitoring of progress [10]. These interventions have shown benefit in the recovery of communication skills over time and continue to be used today.

However, therapy sessions may be compromised by resource accessibility issues, including limited availability of immediate rehabilitation services and trained personnel, or even fragmentation of the therapeutic process due to poor adherence outside clinical settings, This results in diminished patient progress and creates a significant barrier to generalizing learned skills to the patient’s immediate context. This situation may be aggravated by service delivery structures, which often prioritize care in acute and subacute stages, while chronic sequelae may receive less attention, leading to limitations in service continuity and rehabilitation effectiveness [11].

These challenges motivate the exploration of novel strategies to overcome such barriers while also promoting the independent practice of therapeutic activities without geographical constraints. With technological advances and especially the increasing integration of artificial intelligence (AI) in healthcare, new possibilities have emerged to support and enhance the rehabilitation of individuals with aphasia.

Some studies have created pilot predictive models that evaluate language treatment response in aphasia [12,13], and this could favor personalized and efficient therapeutic planning. Other research has focused on utilizing automatic speech recognition to aid in progress monitoring, provide auditory feedback, and assist with word retrieval [14].

This integration of rehabilitation and AI creates a pressing need for evidence-based investigation. Therefore, this study addresses these research gaps through a systematic review of existing randomized controlled trials (RCTs) examining language rehabilitation therapies delivered via digital platforms incorporating AI components to personalize or adapt treatment for individuals with post-stroke aphasia. Additionally, it seeks to determine the effectiveness of these digital interventions compared to conventional therapies, generating robust evidence to guide future clinical decision-making.

## 2. Materials and Methods

### 2.1. Study Protocol and Research Question

A systematic review was conducted following the guidelines of the Cochrane Collaboration Handbook and the Preferred Reporting Items for Systematic Reviews and Meta-Analyses (PRISMA) statement. The protocol was registered with the International Prospective Register of Systematic Reviews (PROSPERO) before formal data extraction began (ID: CRD420251080417).

The research question was structured using the PICO (Population, Intervention, Comparison, Outcomes) framework:

- Population (P): Adults (>18 years) with a clinical diagnosis of aphasia of any type or severity, secondary to a stroke.
- Intervention (I): Language rehabilitation therapies administered via digital platforms (e.g., software, mobile apps, web-based programs) that incorporate AI components to personalize or adapt the treatment (e.g., adaptive algorithms, natural language processing for feedback, machine learning to adjust difficulty).
- Comparison (C): A control group receiving conventional speech-language therapy (therapist-led), digital therapy without AI components, or a wait-list/usual care control (placebo or no intervention).
- Outcomes (O): Changes in language functions measured with standardized scales (e.g., naming, fluency, comprehension), improvements in functional communication in daily life, communication-related quality of life, and the incidence of adverse events.

### 2.2. Eligibility Criteria

The inclusion and exclusion criteria for the selection of studies are detailed in Table 1.

### 2.3. Information Sources and Search Strategy

A systematic search was conducted in the following electronic databases: PubMed/MEDLINE, Scholar, Scopus, Web of Science, Science Direct and Springer. The inclusion of clinical trial registries was considered essential to capture emerging research in this rapidly advancing field. Additionally, the reference lists of included articles and relevant reviews were screened to identify further studies. Reference management was performed using Zotero (version 7.0.23, 2025), and the screening process was managed on the Rayyan platform (web-service, continuously updated).

### 2.4. Search Algorithm

A sensitive search strategy was designed by combining MeSH terms (where applicable) and keywords. Boolean operators (AND, OR) were used.

(aphasia OR dysphasia) AND (stroke OR “cerebrovascular accident”) AND (“digital therapy” OR “computer-based therapy” OR “telerehabilitation” OR “mobile application” OR mHealth) AND (“artificial intelligence” OR “machine learning” OR “adaptive learning” OR “natural language processing”) AND (randomized controlled trial OR randomised controlled trial OR RCT).

### 2.5. Study Selection and Data Extraction

Two independent reviewers (Y.L., L.M.B.) screened the titles and abstracts of the identified records. Potentially eligible articles were reviewed in full text. Discrepancies were resolved through discussion and consensus, or with the mediation of a third reviewer if necessary (J.A.D.V.). Cohen’s Kappa statistic was calculated to measure inter-rater agreement.

A standardized form was used for data extraction, collecting information on:

- Study Characteristics: Author, year, country, design, sample size.
- Population Characteristics: Mean age, sex, aphasia type and severity, time since stroke.
- Intervention Characteristics: Digital platform, AI type, dose (frequency, session duration), total treatment duration.
- Control Group Characteristics: Description of the comparison therapy.
- Outcomes: Quantitative data (mean, SD, sample size) for the outcomes of interest.

**Figure 1:**
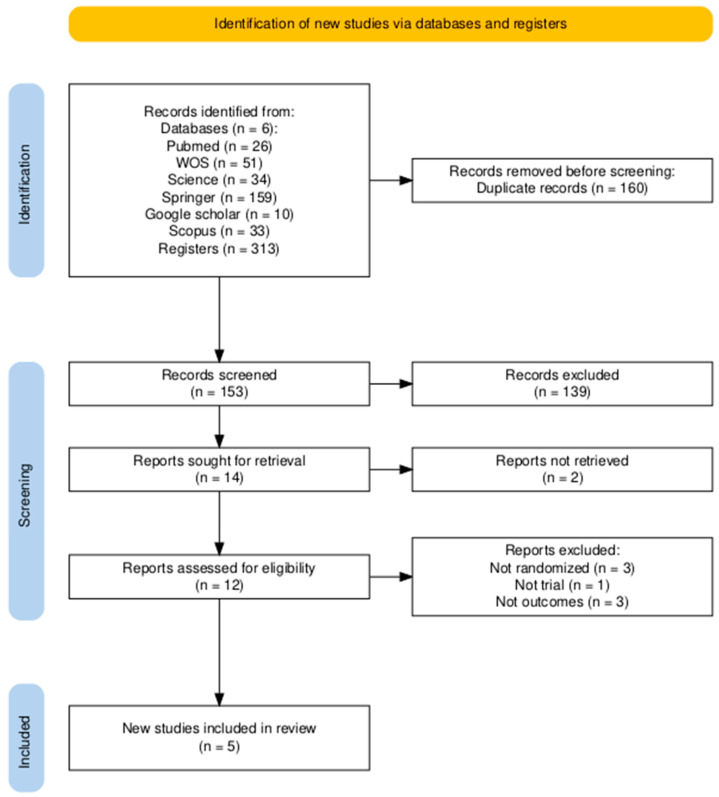
PRISMA flow diagram with the search and study selection strategy. Inter-rater agreement was assessed at different stages: records screening (Cohen’s kappa = 0.80), eligibility assessment (Cohen’s kappa = 0.90), and final inclusion (Cohen’s kappa = 0.95 and 0.82), indicating high levels of agreement between reviewers.

**Figure 2:**
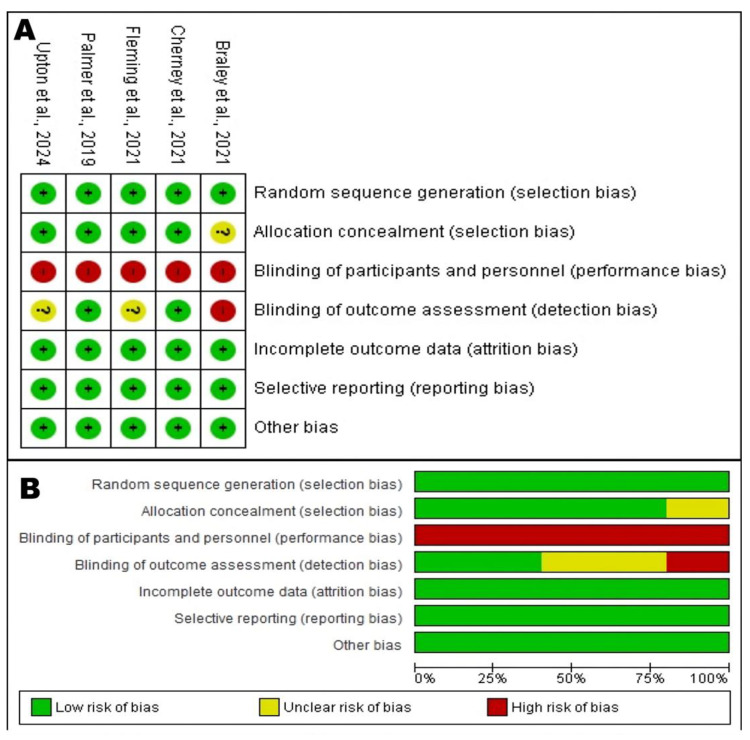
Risk of Bias Assessment of the RCTs. (**A**) Risk of bias summary: Judgments about each risk of bias item for each included study. The “+” symbol (green) indicates a low risk of bias, “?” (yellow) indicates an unclear risk, and “-” (red) indicates a high risk. (**B**) Risk of bias graph: Judgments about each risk of bias item presented as percentages across all included studies [16,17,18,19,20].

Figure 1 was created with the online R package Version 4.5.1 PRISMA2020 [15] (https://estech.shinyapps.io/prisma_flowdiagram/ (accessed on 24 May 2025)). Figure 2 was created using Review Manager version 5.4^®^ (RevMan, The Cochrane Collaboration, accessed on 24 May 2025).

### 2.6. Risk of Bias and Quality of Evidence Assessment

The risk of bias in the included RCTs was assessed by two independent reviewers (Y.L., L.M.B.) using the Cochrane “Risk of Bias 2” (RoB 2) tool. The following domains were evaluated: (a) the randomization process, (b) deviations from intended interventions, (c) missing outcome data, (d) measurement of the outcome, and (e) selection of the reported result.

Additionally, the overall methodological quality of the studies was assessed using the Jadad scale, which assigns a score from 0 to 5. Studies with a score of ≥3 were considered high quality.

To verify the consistency of the evaluation process, a selection of the studies was re-examined. Cohen’s kappa coefficient was calculated using IBM SPSS Statistics (version 27.0, IBM Corp., Armonk, NY, USA; accessed on 24 May 2025) to numerically measure the level of agreement between the reviewers.

### 2.7. Data Synthesis

Due to the significant diversity among the included studies, specifically regarding their interventions, therapeutic approaches, and measured outcomes, a qualitative synthesis was performed. The findings were presented in a narrative format, supplemented by tables that summarize the key characteristics and results of the studies.

### 2.8. Ethical Considerations

Ethical approval was not required for this investigation, as it is a systematic review of previously published literature. The study did not involve any direct interaction with human or animal subjects.

## 3. Results

### 3.1. Study Selection and General Characteristics

The study selection process adhered to the PRISMA (Preferred Reporting Items for Systematic Reviews and Meta-Analyses) guidelines and is detailed in the corresponding flow diagram. The initial search across six electronic databases (Pubmed, WOS, Science, Scopus, Springer, Scholar) and clinical trial registries identified a total of 313 records. After the removal of 160 duplicates, 153 unique records proceeded to the screening phase. During this phase, 139 records were eliminated in the title and abstract filter because they did not meet the preliminary inclusion criteria.

Of the 14 remaining articles, full texts were sought for a more detailed eligibility assessment. It was not possible to retrieve two of these reports (See Figure 1). The remaining 12 full-text articles were rigorously evaluated for inclusion. At this stage, seven articles were excluded for specific reasons: three were not randomized trials, one was not a clinical trial at all, and three did not report outcomes relevant to the objectives of this review. This meticulous selection process culminated in the inclusion of five unique studies that form the basis of this analysis.

The five trials included in this systematic review were conducted in high-income Western countries, reflecting the current epicenter of research and development in this field. Three studies were carried out in the United Kingdom (Upton et al., 2024 [16]; Fleming et al., 2021 [17]; Palmer et al., 2019 [18]), and two in North America (Braley et al., 2021 [19], in the US and Canada; Cherney et al., 2021 [20], in the US). The publication period, spanning from 2019 to 2024, underscores the contemporary nature and rapid evolution of research on digital therapies for aphasia.

The geographical concentration of these studies in settings with robust healthcare systems, such as the UK’s National Health Service (NHS), and well-funded research ecosystems is noteworthy. The development, validation, and implementation of AI-driven digital health technologies require significant investment in technological infrastructure, academic-clinical collaboration, and funding for large-scale trials, such as the multicenter Big CACTUS study. This reality has direct implications for the generalizability of the findings. The studied platforms, developed and tested primarily in English-speaking populations, may have limited applicability in low- and middle-income countries, where barriers related to access to smart devices, internet connectivity, digital literacy, and healthcare infrastructure could impede their widespread adoption without substantial investment in localization and cultural adaptation.

The designs of the included studies show considerable methodological heterogeneity, reflecting the diversity of approaches to evaluating these new interventions. Included were two-arm RCTs (Braley et al., 2021 [19]; Cherney et al., 2021 [20]), a large-scale, three-arm multicenter RCT (Palmer et al., 2019 [18]), a crossover RCT (Fleming et al., 2021 [17]), and a Phase II within-subject design (Upton et al., 2024 [16]). A summary of the general characteristics of the included studies is presented in Table 2.

### 3.2. Participant Characteristics

A total of 366 individuals participated in the five included studies. Sample sizes varied considerably, from 27 participants in the within-subject design study by Upton et al. (2024) [16] to 240 participants in the modified intention-to-treat analysis of the large-scale trial by Palmer et al. (2019) [18]. The mean age of participants was consistently around 60 years (range 58.3 to ~64.9 years), a demographic representative of the stroke population.

A fundamental and unifying methodological feature of all studies was their exclusive focus on individuals with chronic aphasia. The mean time since stroke ranged from 38.1 months (in the control group of Braley et al., 2021 [19]) to 83 months (Upton et al., 2024 [16]). This focus on the chronic phase (>6–12 months post-stroke) is a significant methodological strength. Observed improvements in language function are more likely to be attributed to the therapeutic intervention itself, rather than to the spontaneous biological recovery that characterizes the acute and subacute phases post-stroke. Therefore, the findings of this review are particularly relevant for the long-term management of aphasia, a context in which intensive treatment options are often limited.

The aphasia profiles of the participants showed considerable variability, reflecting the clinical spectrum of the disorder. Some studies focused on specific deficits, such as anomia (word-finding difficulty) in the study by Upton et al. (2024) [16] or auditory comprehension deficits in Fleming et al. (2021) [17]. Others used broader inclusion criteria, such as a Western Aphasia Battery-Revised Aphasia Quotient (WAB-R AQ) score of ≤90 (Braley et al., 2021 [19]), or included a mix of fluent and non-fluent aphasia types (Cherney et al., 2021 [20]). The WAB-R AQ is a standardized and widely used measure to quantify the overall severity of aphasia, where lower scores indicate greater severity. The Big CACTUS trial, the largest in the cohort, stratified participants by the severity of their word-finding difficulty, allowing for a more nuanced analysis.

This clinical heterogeneity, while increasing the applicability of the findings to a real-world patient population, also introduces challenges for direct comparison of results across studies. The effectiveness of a digital intervention may vary depending on the patient’s aphasia profile. For example, a therapy designed to improve word retrieval may have a different impact on an individual with pure anomia compared to one with global aphasia and severe comprehension deficits. Emerging evidence supports this notion; the economic analysis of the Big CACTUS trial suggested that computerized therapy might be more cost-effective for individuals with mild to moderate word-finding difficulties compared to those with severe deficits. This suggests that a “one-size-fits-all” approach to digital therapy may be suboptimal. The future trajectory of this field will likely move towards more personalized medicine, where the type of AI-driven digital therapy is precisely tailored to each patient’s specific neuropsychological and linguistic profile, requiring further research focused on subgroup analyses. Table 3 details the characteristics of the participants in the reviewed studies.

### 3.3. Intervention Details and Comparison Groups

The interventions evaluated in this review represent a spectrum of technological sophistication, from highly adaptive AI-driven platforms to more static programs with remote supervision. This variability is key to understanding the mechanisms underlying the observed outcomes.

At the most advanced end of the spectrum are platforms like Constant Therapy, which uses a proprietary “NeuroPerformance Engine” to personalize exercise selection and adjust task difficulty based on the user’s real-time performance. Similarly,

iTalkBetter employs a “NUVA Classifier” that performs real-time speech analysis to provide immediate feedback. These technologies embody the principle of adaptive therapy, which aims to keep the patient in a zone of “desirable difficulty.” This concept, rooted in the science of learning and neuroplasticity, posits that for lasting learning and neural reorganization to occur, tasks must be challenging enough to stimulate the brain but not so difficult as to cause frustration and abandonment. Adaptive AI engines are designed to dynamically calibrate this level of challenge, thereby optimizing the quality and effectiveness of each practice session.

Other platforms, such as Listen-In and StepByStep (used in the Big CACTUS trial), employ more specific adaptive algorithms. Listen-In adjusts difficulty in word-picture matching tasks, while StepByStep offers personalization and adaptation based on user performance, automatically moving them to higher or lower levels according to their progress. In contrast, the Web ORLA^®^ platform is explicitly non-adaptive; the difficulty of the oral reading tasks is adjusted remotely by a clinician. This model more closely resembles traditional telerehabilitation than an autonomous AI-driven therapy.

The dose and duration of therapy also varied significantly across studies. Intensity, measured in hours per week, ranged from a moderate-intensity regimen of approximately 2.5 h weekly (Braley et al., 2021 [19]) to high-intensity regimens of 7.5 to 8.5 h weekly (Upton et al., 2024 [16]; Fleming et al., 2021 [17]). The total duration of the intervention varied from 6 weeks (Upton et al., 2024 [16]; Cherney et al., 2021 [20]) to 6 months (Palmer et al., 2019 [18]). A key finding is that, regardless of the schedule, the total therapy dose (total time on task) was substantial in all studies, far exceeding the typical provision of outpatient therapy in many healthcare systems. For example, the study by Fleming et al. (2021) [17] delivered an average of 85 h of therapy in 12 weeks, while the Palmer et al. (2019) [18] trial facilitated an average of 28 h of self-managed practice over 6 months.

This data offers a nuanced perspective on the intensity debate in aphasia therapy. While some systematic reviews suggest that higher weekly intensity (>8 h/week) is associated with better outcomes, the studies reviewed here indicate that both short-term, high-intensity regimens (Fleming et al. [17]) and long-term, moderate-intensity regimens (Palmer et al. [18], Braley et al. [19]) can produce clinically significant effects. This suggests that the critical factor may be the total cumulative dose of practice. The main advantage of digital therapy is its ability to facilitate a high total dose in a way that is logistically flexible and more economically viable than traditional face-to-face therapy.

The control groups used were also diverse, ranging from usual care combined with an attention control (puzzles) (Palmer et al., 2019 [18]), to paper-based exercises (Braley et al., 2021 [19]), a placebo game (Bejeweled 2) (Cherney et al., 2021 [20]), or within-subject designs where participants acted as their own control (Fleming et al., 2021 [17]; Upton et al., 2024 [16]). Table 4 summarizes the intervention details.

### 3.4. Synthesis of Efficacy Results

All five included trials reported positive outcomes, demonstrating the efficacy of digital therapies in improving various aspects of language function in people with chronic aphasia. However, the magnitude and nature of these effects varied depending on the intervention and the outcome measure used.

The study by Upton et al. (2024) [16], using the iTalkBetter app, showed a 13% improvement in word retrieval accuracy on a naming test. Crucially, it also observed a transfer of these gains to more functional language, with an increase of 4.4 informative words in spontaneous speech. These improvements were maintained 12 weeks after the therapy ended, suggesting durable learning.

Fleming et al. (2021) [17], evaluating the Listen-In app for auditory comprehension, reported an 11% improvement in accuracy on an auditory comprehension task, which translated to a large effect size (d = 1.12), indicating a substantial clinical impact.

The trial by Braley et al. (2021) [19], using the Constant Therapy platform, yielded one of the most notable results in terms of overall impact. The experimental group showed a 6.75-point improvement on the WAB-R AQ, a measure of overall aphasia severity. This gain was dramatically greater than that observed in the control group, which only improved by 0.38 points, a difference that is both statistically and clinically significant.

Cherney et al. (2021) [20], with the non-adaptive Web ORLA^®^ platform, found more modest but statistically significant improvements in the WAB Language Quotient (WAB-LQ). Interestingly, the gains continued to increase during the follow-up period, rising from +0.99 points immediately post-therapy to +2.70 points at follow-up, which could indicate a learning consolidation effect.

Finally, the Big CACTUS trial by Palmer et al. (2019) [18], the largest in the review, reported the greatest effect on a specific task. The group using the StepByStep computerized therapy improved by 16.4% in retrieving personally relevant words, compared to minimal changes in the usual care (+1.1%) and attention control (+2.4%) groups.

Despite these positive results, a deeper analysis reveals a critical and recurring pattern: the generalization gap. This term refers to the discrepancy between the significant improvements observed in impairment-level tasks (i.e., the ability to perform specific exercises like naming pictures) and the lack of transfer of these gains to functional communication in daily life.

The Big CACTUS trial is the clearest example of this phenomenon. Despite its resounding success in improving word retrieval (its primary impairment-level outcome), the study found no statistically significant improvement in its functional co-primary outcome: communication ability in a conversation, rated by blinded assessors using the Therapy Outcome Measures activity scale. Similarly, no improvements were observed in quality of life or in participants’ perception of communication. This finding is of utmost importance, as it suggests that while AI is an exceptionally effective tool for repetitive practice and the automation of specific language skills, this does not automatically translate into richer or more effective real-world conversations.

The study by Upton et al. (iTalkBetter) [16] offers a promising counterpoint, as it did demonstrate a transfer of gains in naming to an increase in the informational content of connected speech. This could be due to the specific features of the platform, which focuses on high-frequency words and provides real-time feedback on oral production.

The generalization gap represents a fundamental challenge for the field of aphasia rehabilitation. It indicates that future developments in digital therapy must go beyond the simple practice of isolated words. To achieve a truly functional impact, platforms may need to incorporate modules that explicitly train sentence construction, discourse coherence, and conversational strategies. The integration of therapeutic techniques such as Response Elaboration Training (RET), which focuses on expanding patient productions in a back-and-forth context, could be a promising avenue for closing this gap. Table 5 summarizes the quantitative results of the studies.

### 3.5. Methodological Quality and Risk of Bias Assessment

The methodological quality of the included studies was assessed using the Jadad Scale and the Cochrane Risk of Bias tool for randomized trials. The results of these assessments are crucial for determining confidence in the reported findings.

On the Jadad Scale, all five studies scored 3 out of a maximum of 5 (see Table 6). This score, which generally indicates moderate methodological quality, was due to a consistent pattern: all studies were described as randomized and appropriately handled withdrawals and dropouts (1 point for each), but none could be described as double-blind, thus losing points for the description and appropriateness of blinding.

A more detailed analysis, as visualized in Figure 2, reveals a risk of bias profile with clear strengths and weaknesses. In general, the domains of random sequence generation, incomplete outcome data (attrition bias), and selective reporting presented a low risk across all studies. Allocation concealment was also mostly low risk, with only one study rated as unclear. This indicates that the randomization and reporting processes were robust, minimizing the risk of selection and attrition bias.

The most significant and universal methodological limitation in this cohort of studies lies in the blinding of participants and personnel (performance bias). As shown in Figure 2, all five studies were rated at high risk of bias in this domain. This finding should not necessarily be interpreted as a flaw in the study designs, but rather as a challenge inherent to the nature of behavioral interventions. It is practically impossible to blind a participant as to whether they are interacting with a dynamic, gamified, and personalized therapy app, or completing paper-based exercises or playing a simple puzzle game. This knowledge can generate potent expectancy and motivation effects. Participants in the experimental groups may be more engaged, invest more effort, and have a greater belief in the treatment’s efficacy. These non-specific factors alone can lead to better performance, regardless of the app’s therapeutic content. Therefore, the large effect sizes reported should be interpreted with caution, as they likely represent a combination of the intervention’s true therapeutic effect and a non-specific effect driven by differential motivation and engagement between groups.

The blinding of outcome assessment (detection bias) presented a mixed picture. The studies by Cherney et al. (2021) [20] and Palmer et al. (2019) [18] had a low risk of detection bias, as the outcome assessors were blinded to group allocation, which is a major methodological strength that increases confidence in their outcome measurements. Conversely, the study by Braley et al. (2021) [19] was rated at high risk in this domain, which constitutes a notable weakness. An unblinded assessor could, even unconsciously, score the intervention group more favorably, which may have contributed to the large +6.75-point gain on the WAB-AQ. The studies by Upton et al. (2024) [16] and Fleming et al. (2021) [17] were rated as unclear risk due to a lack of explicit information.

The domain of other bias was consistently rated as low risk, suggesting that the studies were free from other significant issues that could skew the results.

## 4. Discussion

### 4.1. Main Findings

The systematic analysis of digital aphasia therapies reveals a compelling narrative of technological promise coupled with significant implementation challenges. The evidence demonstrates that digital interventions represent a paradigm shift in addressing the fundamental dosage gap that has long plagued aphasia rehabilitation. Studies consistently show that these platforms can deliver the high-intensity practice necessary for neuroplastic change, with participants achieving meaningful improvements in language function during the chronic phase of recovery.

The most significant finding is the consistent pattern of positive outcomes across diverse digital platforms. The iTalkBetter app demonstrated a 13% improvement in word retrieval accuracy with notable transfer to spontaneous speech production, evidenced by a 4.4-word increase in informative content that was maintained for 12 weeks post-intervention. Similarly, the Listen-In app produced an 11% improvement in auditory comprehension with a large clinical effect size (d = 1.12), while the Constant Therapy platform yielded a 6.75-point improvement on the Western Aphasia Battery-Revised Aphasia Quotient. These gains are particularly meaningful given that participants were in the chronic phase of aphasia (mean time since stroke: 38.1–83 months), where spontaneous recovery is minimal and therapeutic gains can be attributed directly to the intervention.

The therapeutic success of these digital tools extends beyond individual applications to represent a systematic solution to the research-practice dosage gap. Meta-analyses reveal that evidence-based treatment protocols typically require a median of 20 total hours across 15 sessions, yet standard outpatient care delivers only 7.5 h across 10 sessions. This 2.5-fold disparity explains much of the “voltage drop” observed when research-proven interventions are implemented in clinical practice. Digital platforms uniquely address this gap by providing scalable, accessible, and engaging mechanisms for patients to accumulate the high-dose practice essential for meaningful recovery.

However, the most critical finding is the persistent “generalization gap” between impairment-level improvements and functional communication gains. The Big CACTUS trial exemplifies this challenge, demonstrating a 16.4% improvement in retrieving personally relevant words without corresponding gains in conversational ability or quality of life. This pattern suggests that while digital therapies excel at automating drill-and-practice exercises, they largely fail to address the complex, multi-level language processing required for real-world communication.

### 4.2. Comparison with Previous Literature

The findings align with a growing body of evidence supporting the effectiveness of digital aphasia therapies while revealing critical insights about their limitations. Recent systematic reviews have consistently demonstrated that computer-based and tablet-based self-administered treatments can produce significant improvements in trained language skills, particularly for anomia. Ericson et al. (2025) [21] conducted a comprehensive systematic review of 39 studies involving computer- and smart-tablet-based self-administered treatments for chronic post-stroke aphasia, finding that these interventions were unanimously effective in reducing aphasic symptoms for trained items with good maintenance over time. This finding directly corroborates our analysis that digital platforms successfully address the dosage gap by enabling intensive practice that would be impossible through traditional face-to-face therapy alone.

Cao et al. (2021) [22] conducted a systematic review and meta-analysis of 4 studies on the effects of virtual reality (VR) in aphasia rehabilitation They demonstrated that immersive and motivating experiences promote aphasia recovery through intensive practice, augmented feedback and stimulation of neuroplasticity. Based on the results of our study, we can say that artificial intelligence (AI) could enhance this approach by dynamically adjusting task difficulty, analyzing patient performance and providing more accurate feedback via speech recognition and natural language processing.

The consistency of impairment-level gains across studies is remarkable. Our analysis of effect sizes aligns closely with meta-analytic findings from recent telerehabilitation research. A systematic review and meta-analysis by Cacciante et al. (2021) [23] examining telerehabilitation for people with aphasia found that remote training approaches were as effective as conventional face-to-face treatment, with analysis of 132 participants across five studies showing significant improvements in auditory comprehension and naming performances. This convergent evidence strengthens the conclusion that digital delivery mechanisms do not compromise therapeutic efficacy when properly designed and implemented.

However, the most critical finding from our analysis, the persistent generalization gap, is strongly supported by contemporary literature. The iTalkBetter study (Upton et al., 2024 [16]) provides a notable exception to this pattern, demonstrating successful transfer from trained naming tasks to spontaneous speech production. Participants showed a significant increase of 4.4 informative words in picture description tasks (Cohen’s d = 0.42), representing the first documented case of meaningful functional transfer in digital aphasia therapy trials. This finding is particularly significant because it contradicts the pattern observed in larger trials like Big CACTUS, where intensive digital practice failed to translate to conversational improvements.

The theoretical framework for understanding these disparate outcomes is increasingly well-established. Recent research on AI-assisted aphasia assessment and treatment has provided crucial insights into the mechanisms underlying generalization. Zhong (2024) [24] notes that while AI technologies excel at automating assessment and providing personalized treatment through speech signal processing and pattern recognition, they often fall short in addressing the complex, multi-layered demands of functional communication. This limitation stems from the fundamental difference between practicing discrete language tasks and engaging in dynamic, contextually rich conversation.

The telerehabilitation literature provides additional perspective on the generalization challenge. Systematic reviews have consistently shown that while remote therapy approaches can match traditional therapy in terms of impairment-level gains, the question of functional transfer remains largely unresolved. The recent emphasis on “synchronous” versus “asynchronous” delivery models in telerehabilitation research suggests that the presence of real-time human interaction may be a critical factor in achieving functional outcomes—a component notably absent in most automated digital platforms.

Our analysis also aligns with emerging research on the optimal design principles for digital aphasia interventions. The success of iTalkBetter in achieving functional transfer may be attributable to its sophisticated real-time speech recognition system (NUVA) and adaptive feedback mechanisms, which more closely approximate the dynamic nature of human conversation compared to static picture-naming drills. This finding suggests that the technological sophistication of the platform, particularly its ability to provide contextually appropriate, real-time feedback, may be a critical factor in bridging the generalization gap.

The evidence from recent studies on self-administered digital therapies strongly supports the conclusion that effectiveness depends not merely on the digital delivery method but on the underlying therapeutic strategy being automated. Ericson et al. (2025) [21] found that while most studies focused on word-level interventions, those targeting sentence processing and narrative production showed more promising transfer effects to connected speech. This pattern suggests that the level of linguistic complexity targeted by the intervention is a crucial determinant of functional outcomes.

The comparison with contemporary literature reveals a clear trajectory for the field. While the first generation of digital aphasia therapies successfully demonstrated that automated delivery could match traditional therapy for impairment-level gains, the current generation must address the more complex challenge of functional transfer. The success of iTalkBetter in achieving this transfer, combined with insights from AI and telerehabilitation research, provides a roadmap for developing more sophisticated platforms that can bridge the gap between laboratory gains and real-world communication improvement.

### 4.3. Implications for Therapeutic Strategy and Platform Design

Current digital platforms are very good at delivering repetitive language exercises, but these types of exercises often do not transfer to the situations that matter most: talking with family, participating in social activities, or managing daily tasks. For many people with aphasia, this means that hours of practice may not translate into greater confidence or independence in real-life contexts. The real challenge is to create platforms that go beyond exercises and include therapeutic techniques that reflect natural expressive language. The analysis reveals that the generalization gap is not a technological limitation but a strategic one. Current digital platforms have successfully automated therapeutic approaches that are fundamentally ill-suited for promoting functional transfer. The challenge for the field is not to improve the technology’s ability to deliver drill-and-practice exercises, but to engineer platforms capable of implementing more sophisticated therapeutic strategies designed for generalization [25,26].

RET could emerges as a particularly promising model for addressing this challenge. Unlike traditional naming drills, RET focuses on message construction and utterance elaboration, directly training the skills required for functional discourse. The systematic shaping of patient responses from minimal utterances to complex, multi-clause sentences targets the heart of conversational ability. Digital implementation of RET would require significant technological advancement, including sophisticated speech recognition, natural language processing, and adaptive questioning algorithms, but represents a clear pathway toward functional gains [27,28].

The integration of Verb Network Strengthening Treatment (VNeST) principles offers another strategic direction. By targeting verbs as the syntactic hubs of sentences, digital platforms could strengthen the core linguistic networks that support sentence construction rather than focusing on isolated nouns. This approach has demonstrated superior generalization to both trained and untrained vocabulary, as well as transfer to sentence production, precisely the type of across-level generalization that current platforms fail to achieve [29,30].

### 4.4. Methodological Considerations and Evidence Quality

The interpretation of these positive findings must be tempered by significant methodological limitations. The universal challenge of blinding in behavioral intervention research introduces substantial risk of performance and detection bias. Participants who know they are using an innovative, engaging therapy app may demonstrate enhanced motivation and effort compared to those in control conditions, potentially inflating the apparent efficacy of the intervention. Therefore, the large effect sizes reported should be interpreted with caution, as they likely represent a combination of the intervention’s true therapeutic effect and a non-specific effect driven by differential motivation and engagement between groups.

The heterogeneity across studies further complicates interpretation. Variations in participant characteristics, intervention dose and duration, outcome measures, and methodological quality prevent meaningful quantitative synthesis. This diversity, while reflecting real-world clinical practice, limits the ability to draw definitive conclusions about the optimal parameters for digital therapy delivery.

### 4.5. Global Health Equity and Implementation Challenges

Our findings show that all the digital interventions evaluated were developed and implemented in high-income Western countries, highlighting a significant disparity in research and innovation, and a lack of equity in global health. This concentration, combined with the sophisticated infrastructure required such as reliable connectivity, modern smartphones, and advanced data analytics, limits the applicability of these platforms in low- and middle-income countries (LMICs) [31]. In LMICs, even access to basic resources such as stable electricity, adequate mobile devices, or digital literacy remains uncertain. Furthermore, cultural and linguistic adaptations are essential to ensure these technologies are relevant and effective across different languages and cultural settings [32,33]. This linguistic exclusion is not only a technical limitation of the programs, it is also a violation of the right to inclusive communication, which is especially critical for people with aphasia, whose access to language is already compromised, to mitigate these challenges, not only are technical solutions needed, but also inclusive legislative and policy frameworks aimed at reducing structural digital inequalities, also pilot studies in several languages may be conducted, promoting fair access to therapy and increasing engagement in daily communication.

The digital health literature supports these challenges. Reviews of digital mental health interventions indicate that most studies originate from high-income countries and, although mobile technologies are increasingly available, significant barriers to sustainable implementation in vulnerable contexts remain. Moreover, evaluations of digital tools in resource-limited settings point to critical needs in infrastructure, cost, local adaptability, and technical support [34].

Limited access to trained post-stroke rehabilitation professionals such as speech and language therapists specializing in aphasia and sociocultural barriers also hinder the success of digital therapies unless significant contextual adaptation is undertaken [35]. For instance, few studies address aphasia management in African countries, where challenges range from lack of stroke awareness to limited availability of linguistically appropriate resources [36].

Nonetheless, LMICs also offer unique opportunities for innovation. There is high potential for technological “leapfrogging,” in which regions bypass legacy systems and directly adopt modern, mobile-centered solutions. The widespread adoption of mobile banking platforms such as M-Pesa in Kenya illustrates how mobile-centered ecosystems can thrive and support complementary services, including telehealth applications [37].

Addressing this inequity requires moving away from the traditional model of “exporting finished applications” and instead promoting mobile-first platforms in LMICs. These should be open-source, adaptable, and co-designed with local teams to ensure appropriate cultural, linguistic, and structural customization. This approach fosters local ownership, sustainability, and clinical relevance across diverse contexts [38].

### 4.6. Clinical Implications and Future Directions

The results of these clinical trials demonstrate that AI-assisted digital therapies are complementary tools rather than replacements for traditional therapy. These technologies can significantly increase the therapeutic dose available to individuals with chronic aphasia, reaching between 28 and 85 h of intervention, compared to the conventional 2 to 3 h per week of in-person therapy. This intensive approach aligns with previous evidence showing that interventions such as Constant Therapy, StepByStep, and iTalkBetter lead to notable improvements in word retrieval and auditory comprehension. Clinicians should adopt a strategic approach that leverages the strengths of these platforms while actively addressing their limitations [39].

A persistent limitation noted in the literature is the gap between improvements in language deficit assessments and their transfer to functional communication. Reviews have shown that while automated interventions often enhance specific skills like naming or comprehension, they rarely translate into meaningful improvements in everyday communication [40]. In contrast, only the iTalkBetter app has been reported to facilitate a significant functional transfer, with an increase of +4.4 informative words in spontaneous speech, a gain maintained at 12 weeks (Upton et al., 2024 [16]).

These findings highlight the need to redefine the therapist’s role within a hybrid model. In this paradigm, clinicians shift away from repetitive practice delegated to software and focus instead on delivering more strategic, complex interventions such as RET, VNeST and other complexity-based approaches could be considered as potential strategies to enhance the generalization of language skills complexity-based approaches could be considered as potential strategies to enhance the generalization of language skills. the aim is not just to improve scores on language assessments, but to foster meaningful communication in everyday life and improve overall quality of life for individuals with aphasia [6].

The field now stands at a critical juncture, where technological capabilities are beginning to match therapeutic ambition. By addressing the generalization gap through strategic platform design, improving methodological rigor through innovative trial designs, and expanding access via global health initiatives, digital therapy for aphasia could fulfill its promise of delivering effective, accessible, and equitable rehabilitation for all individuals with aphasia.

## 5. Conclusions

This systematic review concludes that AI-assisted digital therapies are effective, yet their efficacy is highly specific when compared to conventional therapy. These digital platforms successfully address the dosage limitations of traditional care, proving highly effective at delivering intensive practice that improves isolated language impairments such as word retrieval. However, their effectiveness is critically constrained by a consistent failure to generalize these impairment-level gains to functional, everyday communication. This evidence guides future clinical decision-making toward a hybrid model: AI-assisted therapies should be leveraged as powerful complementary tools to automate high-volume drills, which in turn allows clinicians to dedicate their expertise to implementing the complex, context-rich strategies necessary to bridge the gap between skill acquisition and its functional use in the real world.

## Figures and Tables

**Table 1:** Selection criteria.

Criteria	Inclusion	Exclusion
Study Design	RCTs, including parallel, crossover, or cluster-randomized designs.	Systematic reviews, meta-analyses, letters to the editor, commentaries, editorials; non-randomized studies, case series, case reports; clinical trial protocols; conference abstracts.
Type of Intervention	Digital therapies that explicitly use AI algorithms for aphasia rehabilitation. This includes systems that personalize tasks, dynamically adjust difficulty, or provide automated, intelligent feedback.	Therapies that only use software or computers as a presentation tool without AI components; interventions not focused on language rehabilitation; co-administered pharmacological therapies not applied to the control group.
Outcomes	Studies reporting at least one of the following quantitative outcomes: Language Function: Scores on standardized tests (e.g., Western Aphasia Battery-AQ, Boston Naming Test, Token Test). Functional Communication: Scores on functional assessment scales (e.g., ASHA FACS, CETI). Quality of Life: Scores on specific questionnaires (e.g., SAQOL-39).	Studies that only report qualitative, descriptive, or platform usability metrics without clinical outcomes. Studies that do not provide sufficient data for analysis (e.g., mean and standard deviation).
Population/Context	Adults (≥18 years) with a diagnosis of post-stroke aphasia (ischemic or hemorrhagic), at any stage of chronicity (acute, subacute, chronic).	Patients with aphasia caused by traumatic brain injury, brain tumors, neurodegenerative diseases, or other non-vascular etiologies. Patients with severe neurological or psychiatric comorbidities that would prevent participation.
Language	Studies published in English or Spanish.	Other languages without a full translation available.
Publication Period	Articles published between January 2010 and December 2024, to capture contemporary research in AI.	Publications before 2010.

RCTs: Randomized Controlled Trials; AI: Artificial Intelligence; AQ: Aphasia Quotient; ASHA FACS: American Speech-Language-Hearing Association Functional Assessment of Communication Skills for Adults; CETI: Communicative Effectiveness Index; SAQOL-39: Stroke and Aphasia Quality of Life Scale-39.

**Table 2:** General Characteristics of Included Studies.

Study (Author, Year)	Country	Design	N (Total/Groups)	Primary Outcome
Upton et al., 2024 [16]	United Kingdom	Phase II, within-subject	27	% Accuracy with respect to
				# of informative words
Fleming et al., 2021 [17]	United Kingdom	Crossover RCT	35	% Accuracy ACT
Braley et al., 2021 [19]	USA & Canada	Virtual RCT	32 (17 Exp/15 Ctrl)	Change in WAB-AQ
Cherney et al., 2021 [20]	USA	Pilot, single-blind RCT	32 (19 Exp/13 Ctrl)	Change in WAB-LQ
Palmer et al., 2019 [18]	United Kingdom	Multicenter, single-blind, 3-arm RCT	240 (CSLT = 83; UC = 86; AC = 71)	% Word retrieval

Abbreviations: RCT = Randomized Controlled Trial; with respect to = Word Retrieval Test; ACT = Auditory Comprehension Test; WAB-AQ = Western Aphasia Battery-Aphasia Quotient; WAB-LQ = Western Aphasia Battery-Language Quotient; CSLT = Computerized Speech and Language Therapy; UC = Usual Care; AC = Attention Control.

**Table 3:** Study Participant Characteristics.

Study (Author, Year)	N (Total/Groups)	Mean Age (SD)	Aphasia Type	Time Post-Stroke (Months)
Upton et al., 2024 [16]	27	62 (12)	Chronic with anomia, intact repetition	83 (67)
Fleming et al., 2021 [17]	35	61 (12)	Chronic with auditory comprehension deficit	76 (59)
Braley et al., 2021 [19]	32 (17 Exp/15 Ctrl)	Exp: 58.9 (10); Ctrl: 64.2 (9.9)	WAB-R AQ ≤ 90 (indicating moderate severity)	Exp: 53 (56); Ctrl: 38.1 (32)
Cherney et al., 2021 [20]	32 (19 Exp/13 Ctrl)	Exp: 58.3 (13.6); Ctrl: 55.2 (11.5)	Mixed fluent and non-fluent	Exp: 39.8 (40.8); Ctrl: 61.0 (30.2)
Palmer et al., 2019 [18]	240 (CSLT = 83; UC = 86; AC = 71)	~64.9 (13.0)	Chronic with word-finding difficulty; stratified	CSLT: 2.9 (2.9) years; UC: 2.8 (2.6); AC: 3.6 (4.8)

Abbreviations: SD = Standard Deviation; Exp = Experimental Group; Ctrl = Control Group; WAB-R AQ = Western Aphasia Battery-Revised Aphasia Quotient; CSLT = Computerised Speech and Language Therapy; UC = Usual Care; AC = Attention Control.

**Table 4:** Intervention Details and Comparison Groups.

Study (Author, Year)	Digital Platform	AI Type/Adaptation	Dose/Duration	Control Group
Upton et al., 2024 [16]	iTalkBetter App	NUVA Classifier + adaptive therapy	45 h in 6 wks (mean)	Untrained items + no-therapy block
Fleming et al., 2021 [17]	Listen-In App on tablet	Adaptive algorithm in word-picture matching	85 h in 12 wks (80 min/day)	Same group on usual care
Braley et al., 2021 [19]	CT-Research App	Adaptive NeuroPerformance Engine	≥30 min/day, 5 days/wk, 10 wks	Paper-based exercises
Cherney et al., 2021 [20]	Web ORLA^®^ on laptop	Non-adaptive intrinsic, remote adjustment by clinician	90 min/day, 6 days/wk, 6 wks	Placebo game (Bejeweled 2)
Palmer et al., 2019 [18]	StepByStep on computer	Personalization + user-based adaptation	20–30 min/day, 6 months	UC (standard) & AC (hobbies)

Abbreviations: AI = Artificial Intelligence; wks = weeks; min = minutes; h = hours; UC = Usual Care; AC = Attention Control.

**Table 5:** Summary of Primary Outcomes and Comparative Effects.

Study (Author, Year)	Primary Outcome	Quantitative Results
Upton et al., 2024 [16]	% Accuracy with respect to	+13% (SD = 11.7), n = 27
	# of informative words	+4.4 words, n = 27
Fleming et al., 2021 [17]	% Accuracy ACT	+11% (d = 1.12), n = 35
Braley et al., 2021 [19]	Change in WAB-AQ	Exp: +6.75 pts; Ctrl: +0.38 pts
Cherney et al., 2021 [20]	Change in WAB-LQ	Post: +0.99 pts (SD = 1.41); Follow-up: +2.70 pts (SD = 1.01)
Palmer et al., 2019 [18]	% Word retrieval	CSLT: +16.4% (SD = 15.3); UC: +1.1% (11.2); AC: +2.4% (8.8)

Abbreviations: with respect to = Word Retrieval Test; ACT = Auditory Comprehension Test; WAB-AQ = Western Aphasia Battery-Aphasia Quotient; WAB-LQ = Western Aphasia Battery-Language Quotient; SD = Standard Deviation; Exp = Experimental Group; Ctrl = Control Group; CSLT = Computerised Speech and Language Therapy; UC = Usual Care; AC = Attention Control; pts = points.

**Table 6:** Jadad Scale Score.

Study	1. Described as Randomized? (Yes = 1)	2. Appropriate Randomization Method? (Yes = 1)	3. Described as Double-Blind? (Yes = 1)	4. Appropriate Blinding Method? (Yes = 1)	5. Description of Withdrawals/Dropouts? (Yes = 1)	Total Jadad Score (/5)
Upton et al., 2024 [16]	1	1	0	0	1	3
Fleming et al., 2021 [17]	1	1	0	0	1	3
Braley et al., 2021 [19]	1	1	0	0	1	3
Cherney et al., 2021 [20]	1	1	0	0	1	3
Palmer et al., 2019 [18]	1	1	0	0	1	3

## Data Availability

Data are contained within the article.

## Abbreviations

The following abbreviations are used in this manuscript:

ACT	Auditory Comprehension Test
AI	Artificial Intelligence
AOM	Acute Otitis Media
ASHA	American Speech-Language-Hearing Association
BDAE	Boston Diagnostic Aphasia Examination
CETI	Communicative Effectiveness Index
CONSORT	Consolidated Standards of Reporting Trials
CSLT	Computerised Speech and Language Therapy
CVA	Cerebrovascular Accident
FACS	Functional Assessment of Communication Skills for Adults
fMRI	functional Magnetic Resonance Imaging
GBD	Global Burden of Diseases, Injuries, and Risk Factors Study
GM	Grey Matter
NHS	National Health Service
NUVA	Naming Utterance Verifier for Aphasia
ORLA	Oral Reading for Language in Aphasia
PICO	Population, Intervention, Comparison, Outcomes
PLORAS	Predicting Language Outcome and Recovery After Stroke
PRISMA	Preferred Reporting Items for Systematic Reviews and Meta-Analyses
PROSPERO	International Prospective Register of Systematic Reviews
PWA	People With Aphasia
RCT	Randomized Controlled Trial
RET	Response Elaboration Training
RoB	Risk of Bias
SAQOL	Stroke and Aphasia Quality of Life Scale
SLT	Speech and Language Therapy
sMRI	structural Magnetic Resonance Imaging
SPD	Spoken Picture Description
VNeST	Verb Network Strengthening Treatment
VR	Virtual Reality
WAB	Western Aphasia Battery
WAB-AQ	Western Aphasia Battery-Aphasia Quotient
WAB-LQ	Western Aphasia Battery-Language Quotient
WAB-R	Western Aphasia Battery-Revised
WM	White Matter
with respect to	Word Retrieval Test
